# Comparative systeomics to elucidate physiological differences between CHO and SP2/0 cell lines

**DOI:** 10.1038/s41598-022-06886-1

**Published:** 2022-02-28

**Authors:** Deniz Demirhan, Amit Kumar, Jie Zhu, Pi Camilla Poulsen, Natalia I. Majewska, Yinong Sebastian, Raghothama Chaerkady, Wen Yu, Wei Zhu, Li Zhuang, Punit Shah, Kristen Lekstrom, Robert N. Cole, Hui Zhang, Michael J. Betenbaugh, Michael A. Bowen

**Affiliations:** 1 Department of Natural Sciences, Acibadem Mehmet Ali Aydınlar University, Istanbul, Turkey; 2 Department of Chemical and Biomolecular Engineering, Johns Hopkins University, Baltimore, MD USA; 3 Cell Culture and Fermentation Sciences, BioPharmaceuticals Development, R&D, AstraZeneca, Gaithersburg, MD USA; 4 Protein Science, Antibody Discovery and Protein Engineering, R&D, AstraZeneca, Gaithersburg, MD USA; 5 Translational Science, AstraZeneca, Gaithersburg, MD USA; 6 Informatics, Data Science and Artificial Intelligence, R&D, AstraZeneca, Gaithersburg, MD USA; 7 BERG, Framingham, MA 01701 USA; 8 Mass Spectrometry and Proteomics Facility, Johns Hopkins University School of Medicine, Baltimore, MD USA; 9 Department of Pathology, Johns Hopkins School of Medicine, Baltimore, MD USA; 10 Allogene Therapeutics, South San Francisco, CA USA

**Keywords:** Proteomics, Animal biotechnology

## Abstract

Omics-based tools were coupled with bioinformatics for a systeomics analysis of two biopharma cell types: Chinese hamster ovary (M-CHO and CHO-K1) and SP2/0. Exponential and stationary phase samples revealed more than 10,000 transcripts and 6000 proteins across these two manufacturing cell lines. A statistical comparison of transcriptomics and proteomics data identified downregulated genes involved in protein folding, protein synthesis and protein metabolism, including PPIA-cyclophilin A, HSPD1, and EIF3K, in M-CHO compared to SP2/0 while cell cycle and actin cytoskeleton genes were reduced in SP2/0. KEGG pathway comparisons revealed glycerolipids, glycosphingolipids, ABC transporters, calcium signaling, cell adhesion, and secretion pathways depleted in M-CHO while retinol metabolism was upregulated. KEGG and IPA also indicated apoptosis, RNA degradation, and proteosomes enriched in CHO stationary phase. Alternatively, gene ontology analysis revealed an underrepresentation in ion and potassium channel activities, membrane proteins, and secretory granules including Stxbpt2, Syt1, Syt9, and Cma1 proteins in M-CHO. Additional enrichment strategies involving ultracentrifugation, biotinylation, and hydrazide chemistry identified over 4000 potential CHO membrane and secretory proteins, yet many secretory and membrane proteins were still depleted. This systeomics pipeline has revealed bottlenecks and potential opportunities for cell line engineering in CHO and SP2/0 to improve their production capabilities.

## Introduction

Mammalian expression systems are the predominantly used platforms for FDA-approved drugs and biologics at pre-clinical and clinical stages^1^. Enhanced production of biotherapeutic proteins is critical for pharmaceutical companies to offer efficacious and affordable drugs to patients. Chinese hamster ovary (CHO) cell lines are the most widely used production hosts compared to other cell lines, including mouse myeloma (SP2/0), baby hamster kidney (BHK-21), murine myeloma (NS0), and human embryonic kidney (HEK)^1,2^. The implementation of stable cell lines is integrated together with bioprocess optimization, medium development, and possibly additional cell line engineering in order to achieve efficient growth rates, along with high productivities, product yields and quality appropriate for scale up and production of clinical grade biologics^3^.

Recent advances in omics technologies have been one avenue to elucidate biological changes in mammalian expression systems that may be relevant to recombinant protein production. Genetic sequencing of the CHO cell line and the Chinese hamster^4–6^ have set the foundation of a systems biology era for understanding CHO cell line physiology for enhanced protein production^7^. Proteomic, transcriptomic and microRNAomic^8,9^ technologies have also been applied to quantitatively track the changes in protein, mRNA and microRNA levels between cell lines exhibiting different growth rates and productivities in order to increase our knowledge about production hosts. Coupling this information with cell line engineering and media development technologies not only enhances mammalian growth and productivity but also ushers in a new era for understanding the cellular properties that may make a particular host line appropriate for production for each molecule of interest.

Another emerging approach that can help improve our understanding of complex biological pathways and their roles in bioproduction is the application of systeomics. Systeomics, in biomedicine, is the integration of proteomics, transcriptomics and genomics data, often at the pathway level, for the discovery of novel gene targets and biomarkers for various diseases^10^. Similar methods can be used for the systematic identification of depleted and enriched pathways in mammalian expression platforms^11,12^ to help identify the bottlenecks and limitations related to cell growth, production yields and product quality.

Although both the CHO and SP2/0 cell lines are commonly used host platforms in industry, they exhibit different cellular characteristics. Due to its natural secretory properties, SP2/0 can often generate high mAb titers. On the other hand, CHO cell lines are the primary expression systems in the $113 billion pharmaceutical market due to their manufacturability, high growth profiles, and glycosylation capability^13^. In order to better understand the overall cellular characteristics and the underlying reasons for the expression behavior in these widely used cell lines, we performed a comparative systeomics analysis on the exponential and stationary phases of an AstraZeneca production cell line called M-CHO and a SP2/0 cell line. Understanding the advantages and disadvantages of these two cell lines can provide us the gene or pathway targets that may be appropriate for cell engineering of superior host platforms. Transcriptomic and proteomic experiments were performed in which Illumina HiSeq was used for mRNA sequencing, and filter aided sample preparation (FASP) technique was coupled with two-dimensional LC–MS to reach deep sequencing at the protein level. This is, to our knowledge, the first comprehensive proteomic study done comparing and contrasting SP2/0 and suspension CHO cell lines.

**Figure 1:**
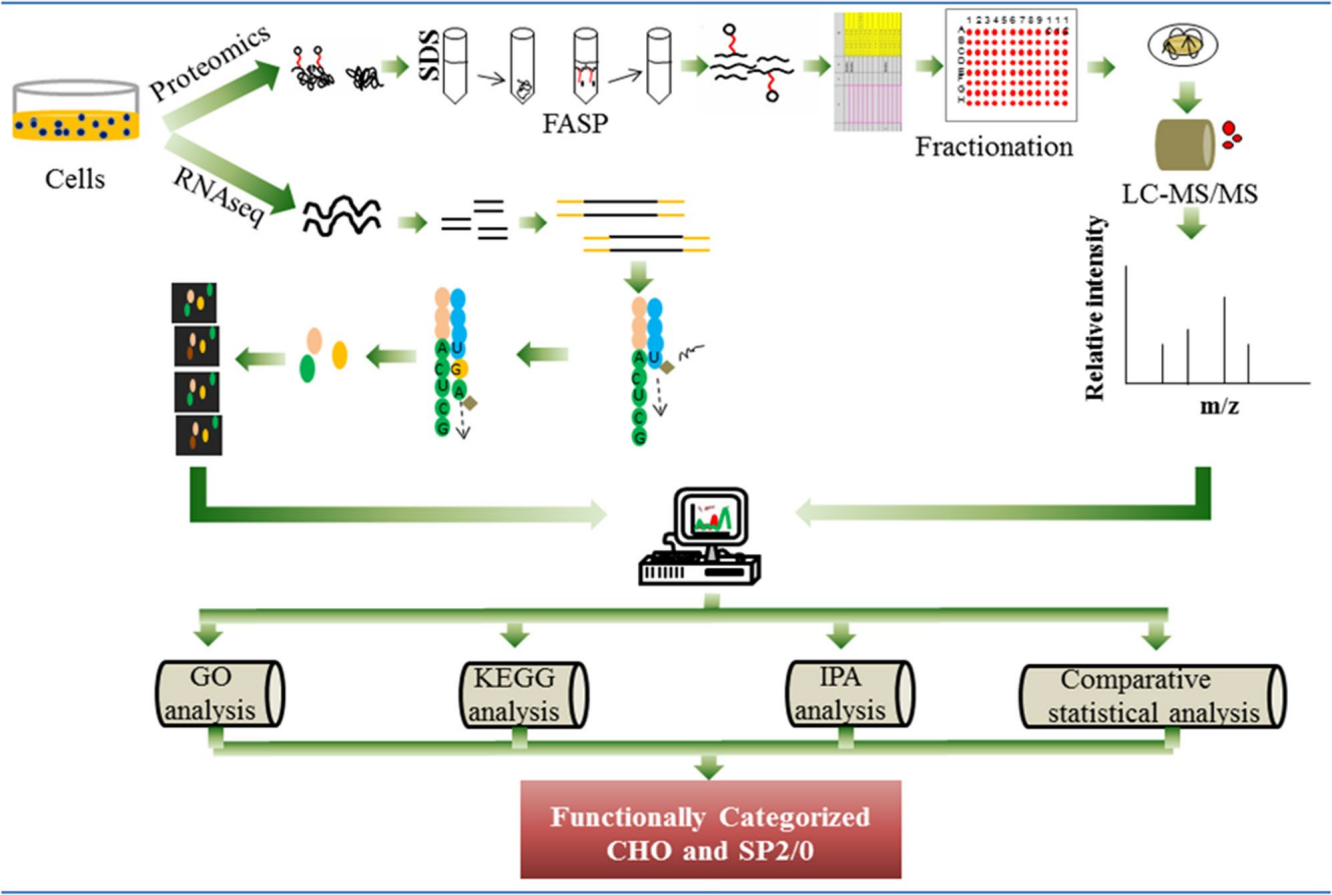
The experimental and bioinformatical workflow of SP2/0 and CHO cell line functional categorization.

Furthermore, to elucidate the differences across CHO cell lines, a CHO-K1 adherent cell line was compared to an M-CHO (proprietary AstraZeneca CHO cell line) suspension cell line using RNAseq and proteomic data^11,12,14^. For the functional annotation, gene ontology (GO) analysis was used, whereas Kyoto Encyclopedia Genes and Genome (KEGG) and Ingenuity Pathway Analysis (IPA) were used to statistically compare the metabolic, signaling and cellular processes at a systems biology level, schematically illustrated in Fig. 1, across the different production hosts and at different phases of the cell growth cycle. The data from the proteomic and transcriptomic analyses was then subjected to comparative analysis at the gene level along with a comparative systeomics approach. From these studies, we identified 10,500 and 13,500 transcripts for M-CHO and SP2/0 respectively. Deep proteomic coverage also yielded 7118 and 7410 identified proteins for SP2/0 and CHO cell lines, respectively.

It will be helpful for future cell designers and bioproduction engineers to understand the capabilities of CHO versus SP2/0 by analyzing omics data. To our knowledge, this is the first large scale study that reveals the specific differences at the gene expression, protein, and pathway level of two of the most widely used mammalian expression systems. This analysis can then serve as the basis for understanding the advantages and disadvantages of these critical production hosts as well as suggesting opportunities to improve their capabilities and capacities in the future through genetic intervention or process design.

## Results

Recent advances in both sequencing and mass spectrometry technologies have enabled the generation of high quality data sets about expression systems such as Chinese hamster ovary (CHO) and others^11,15,16^. In order to obtain even greater insights from these data sets, an emerging approach called comparative systeomics was used in this study to analyze whole cell proteomic and transcriptomics data of CHO and SP2/0. Firstly, a comprehensive omics study was performed on both exponential and stationary phases of two model cell lines, M-CHO and SP2/0, in order to evaluate differences in their proteome and transcriptome expression patterns, as well as the changes in each cell line between the exponential and stationary phases (see Fig. 1). In order to increase the solubilization of whole cell proteins, including membrane proteins, the filter aided sample preparation (FASP) method was used^17^, and high and low pH reversed phase liquid chromatography was coupled prior to MS/MS to substantially increase the proteome coverage^18^. Digests from the exponential phase were separated into 24 fractions and ran twice on LC/MS/MS, whereas the digests from stationary phase were separated into 48 fractions prior to LC/MS/MS analysis. Interestingly, separation into 24 and 48 fractions identified a similar number of proteins, as shown in Table 1, suggesting that separating the lysate into 24 fractions with duplicate runs can be sufficient to reach high numbers of identified proteins. A box plot justifying the same amount of protein was injected to the LC/MS/MS is given in Supplementary Fig. S2.

**Table 1:** Results of RNAseq and proteomics analysis for CHO and SP2/0 cell lines.

Cell lines analyzed	mRNA identified	Proteins identified	Number of unique peptides	Average number of peptides per protein
M-CHO exponential	10,613	6949	56,558	~ 8
M-CHO stationary	10,641	6539	45,961	~ 7
SP2/0 exponential	13,675	6110	47,669	~ 8
SP2/0 stationary	13,727	6800	53,324	~ 8
CHO-K1 ATCC stationary	10,669	6358	52,019	~ 8

RNAseq resulted in the identification and quantification of more than 10,500 transcripts for M-CHO, whereas the sequencing of SP2/0 identified and quantified more than 13,500 transcripts, likely due to the superior annotation of the mouse genome. The identified mRNA, along with their normalized values and triplicates belonging to M-CHO exponential, M-CHO stationary, and CHO-K1 stationary phases are tabulated in Supplementary Table 1, whereas Supplementary Table 2 includes the mRNA values measured for SP2/0 exponential and SP2/0 stationary phases. Analogously, label free proteomic experiments resulted in the identification of 45,000–55,000 unique peptides belonging to the 6000–7000 grouped proteins with a 1% FDR (false discovery rate) for both peptides and proteins. The average number of peptides identified per protein was around 7–8, providing high coverage for most proteins. This represents a whole deep sequencing proteomic profiling of SP2/0 and a serum free suspension CHO cell line, yielding 7118 and 7410 identified proteins for SP2/0 and CHO cell lines, respectively. A previous analysis of MS/MS spectra of a serum-bearing and attachment-dependent model CHO-K1 cell line by our group identified 6358 proteins using the same search criteria^11^, and another analysis of two CHO cell lines (CHO-S and CHO DG44) identified 9359 unique proteins^12^. The protein and peptide information belonging to M-CHO exponential, M-CHO stationary, SP2/0 exponential, SP2/0 stationary and a control CHO-K1 ATCC stationary are compiled in Supplementary Tables 3, 4, 5, 6, and 7, respectively, with a summary of these results in Table 1.

### Correlation and comparison of CHO and SP2/0 proteomes and transcriptomes

**Figure 2:**
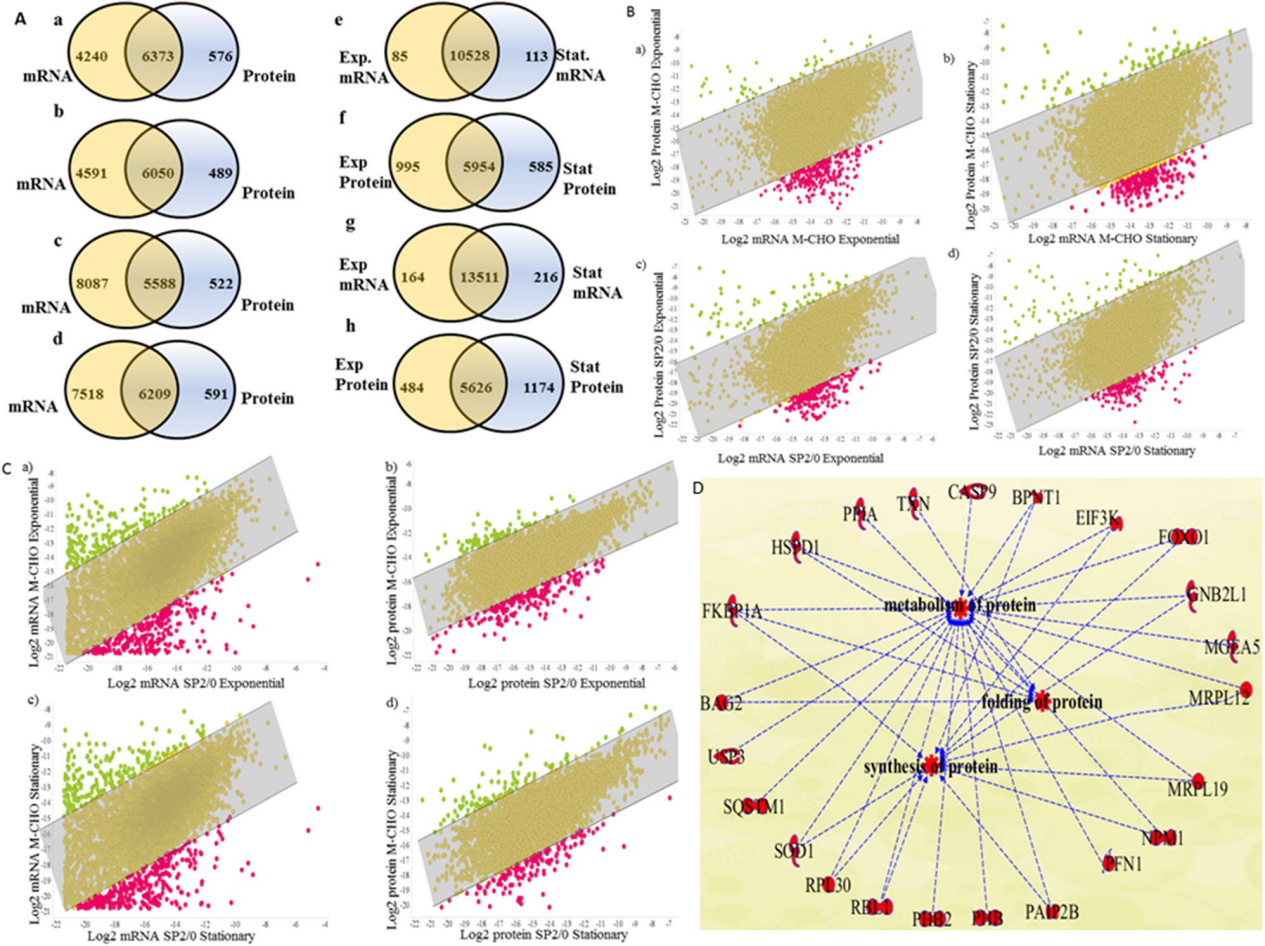
(**A**) Number of genes identified in mRNA and/or protein levels (a) M-CHO exponential phase mRNA versus protein comparison (b) M-CHO stationary mRNA versus protein comparison (c) SP2/0 exponential mRNA versus protein comparison (d) SP2/0 stationary mRNA versus protein comparison (e) M-CHO exponential versus stationary mRNA comparison (f) M-CHO exponential and stationary protein comparison (g) SP2/0 exponential and stationary mRNA comparison (h) SP2/0 exponential and stationary protein comparison (**B**) (a–d) Comparison and correlation of mRNA and protein data in M-CHO or SP2/0 cell lines in exponential and/or stationary phases (**C**) (a–d) Comparison and correlation of mRNA and protein data between the M-CHO and SP2/0 cell lines in exponential or stationary phases (**D**) Downregulated proteins in M-CHO cells compared to SP2/0 cells.

Due to the lack of comprehensive omics data sets, little is known regarding the differences in proteome and transcriptome expression patterns between CHO and SP2/0 or about the changes between the exponential and stationary phases of these cells at protein or mRNA levels. In order to perform a comprehensive comparison, mRNA and protein levels of data sets were compared between M-CHO and SP2/0 cell lines along with different phases. A standard normalized FPKM (fragments of reads mapped per kilobase of exon model) was used to correlate and compare the mRNA values of the samples, whereas the abundance level across the proteins and between the samples was compared using the normalized spectral abundance factor (NSAF), accounting for the length of the identified proteins^19^. Firstly, the genes having both mRNA and protein expression were mapped for both cell lines under the two conditions, resulting in 5500–6000 genes exhibiting both mRNA and protein expression in the separate phases (exponential and stationary) for each cell line (Fig. 2Aa–d). An additional 4000–8000 genes were identified and quantified only in the mRNA transcripts for each cell line, while 500–600 additional genes were found only in the proteome data. An alternative evaluation examined which of these genes were found in both the exponential and stationary phases for transcriptomics and proteomic data for each cell lines, as shown in Fig. 2Ae–h. Over 10,000 genes were identified from transcriptomics data in both exponential and stationary phases for each cell line, while more than 5500 genes were elucidated in the proteome of each cell type for both phases.

Next, pair-wise comparisons were performed; as indicated in Figs. S1 and S2, the relative expression levels between the two phases were similar for both SP2/0 and M-CHO cells. Secondly, protein and mRNA expression levels were compared for each phase of growth in each cell line in Fig. 2B, while the mRNA and protein expression levels were compared between the two cell lines in Fig. 2C on a logarithmic scale. The confidence level calculations illustrated that a majority of the genes were found to be in the 90 or 95% confidence interval. Examples of groups of genes that lie outside the 95% confidence interval are shown in Fig. 2D for the case of stationary phase proteomics comparison between the SP2/0 and M-CHO cell lines. These groups, which are at least 1.8-fold downregulated in M-CHO cells compared to the SP2/0 cells based on NSAF, are associated with protein folding, protein synthesis and protein metabolism. For example, PPIA-cyclophilin A, known to accelerate protein folding, and HSPD1, which plays a role in protein folding and assembly, are lower in CHO cell lines during the stationary phase. Also, the translation initiation factor, EIF3K displayed a lower NSAF value in M-CHO cells. Interestingly, co-expression of translation initiation factors such as EIF4A was previously shown to increase the expression of an antibody more than 3–fourfold in one mammalian cell line (COS)^20^. The growth curves of these two cell lines can be found in Figs. S3a,b and S4.

In addition to the cell cycle and protein folding pathways, apoptosis and actin cytoskeleton signaling pathways were found to be differentially expressed between the two cell lines. The actin cytoskeleton expression was found to be lower in the exponential phase SP2/0 proteome data relative to exponential phase in M-CHO. Interestingly, the actin cytoskeleton was also found to be a biological hub, providing crosstalk with PAK and RAC signaling (Supplementary Figs. S5 and S6). Previous research has shown that destabilizing the actin cytoskeleton with either MTX or Cytochalasin D in CHO cells can increase the production of recombinant secreted alkaline phosphatase by 50–150 fold^21^.

### Pathway analysis of CHO and SP2/0 cell lines

In order to further explore differences between CHO and SP2/0 cell lines at the systems level, we applied pathway analytical tools, including KEGG and IPA, along with biological, molecular and cellular functional analysis tools such as GO (Fig. 1). Both CHO (M-CHO and CHO-K1) and SP2/0 RNAseq and proteome data were mapped to the *Criteculus griseus* and *Mus musculus* KEGG identifiers and pathways, respectively, with enrichment and depletion analyses performed using a hypergeometric distribution. The p-value results from both these tests are listed in Supplementary Table 8 with CHO-K1 data included to determine whether the results vary across different CHO cell lines. In this analysis, we focused on (1) comparing the enrichment and depletion results of stationary and exponential phases for both cell lines (2) comparing the over-represented and under-represented pathways for the M-CHO, SP2/0, and CHO-K1 ATCC cell lines. When the hypergeometric distribution test was applied to compare exponential and stationary phases, whole proteomics and transcriptomics *p*-values indicated that several pathways, such as apoptosis, RNA degradation, and proteasome, exhibited a higher representation in the CHO stationary phase. In addition, analyzing the proteomics for both exponential and stationary phases increased the number of proteins identified in the CHO proteome compared to previous studies^3,11,22^. For instance, proteins such as TNFSF10 (TRAIL) from the apoptosis pathway, EDEM1, CRYAB, and Mbtps1 from the protein processing pathway were shown to be expressed in the current M-CHO study. Other proteins, such as ERGL and S2P involved in protein processing pathways, were identified in SP2/0 cells in this study even though they were absent from the CHO proteome.

**Figure 3:**
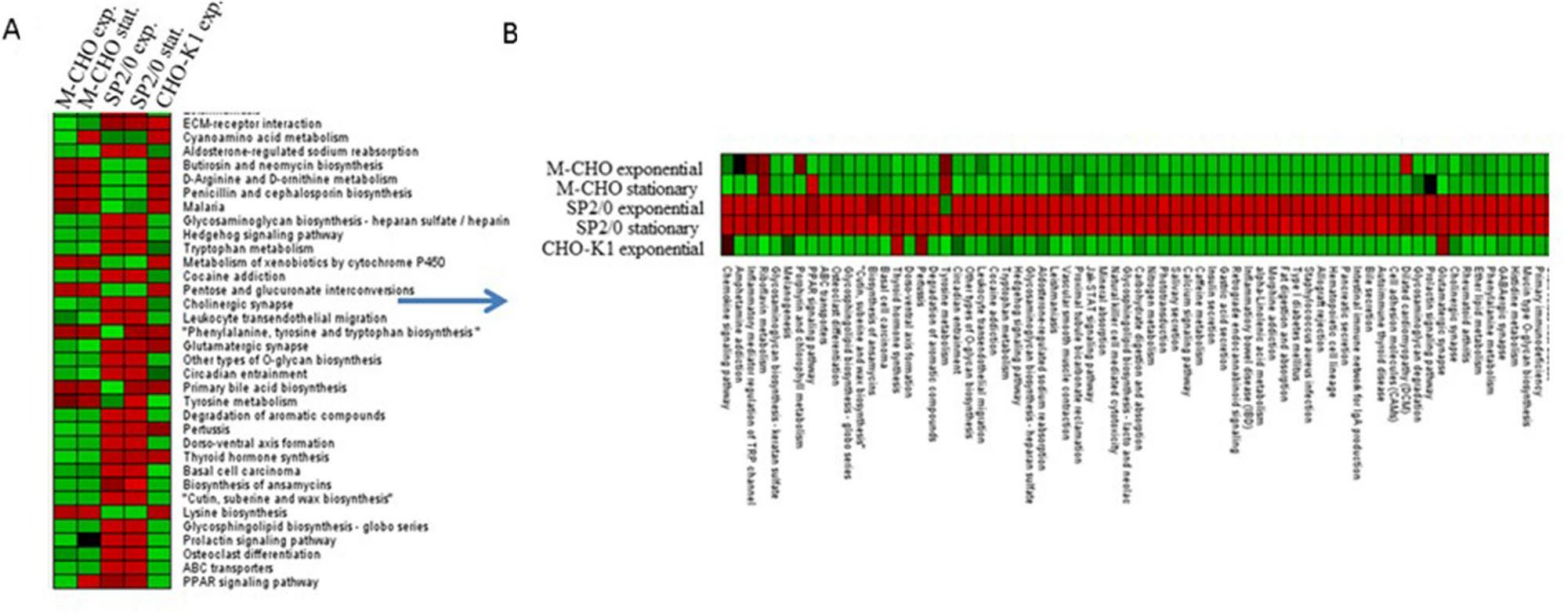
(**A**) Heatmap of* p*-values from hypergeometric analysis of exponential and stationary phase M-CHO and SP2/0 cell lines and exponential phase CHO-K1 cell line (**B**) Heatmap of selected pathways from which are upregulated in SP2/0 exponential and SP2/0 stationary.

Shown in Fig. 3A is a heatmap that illustrates the proteomics changes in pathway depletion p-values for the exponential and stationary phases of M-CHO, SP2/0 and CHO-K1 stationary phase as a control. In all three cell lines, ribosome, RNA-transport, and spliceosome were found to be the highest enriched pathways, whereas metabolic pathways such as glycerolipid and glycerophospholipid metabolism were found to be depleted in CHO cells compared to the SP2/0 cells. The shared 288 pathways between CHO and SP2/0 cells were further investigated. The overall number of pathways showing significant depletion in CHO cells was higher in number compared to SP2/0 cells. Retinol metabolism was the only group showing slight under-representation in SP2/0 cells for both phases compared to M-CHO cells while all others groups were over-represented in SP2/0 compared to CHO.

**Figure 4:**
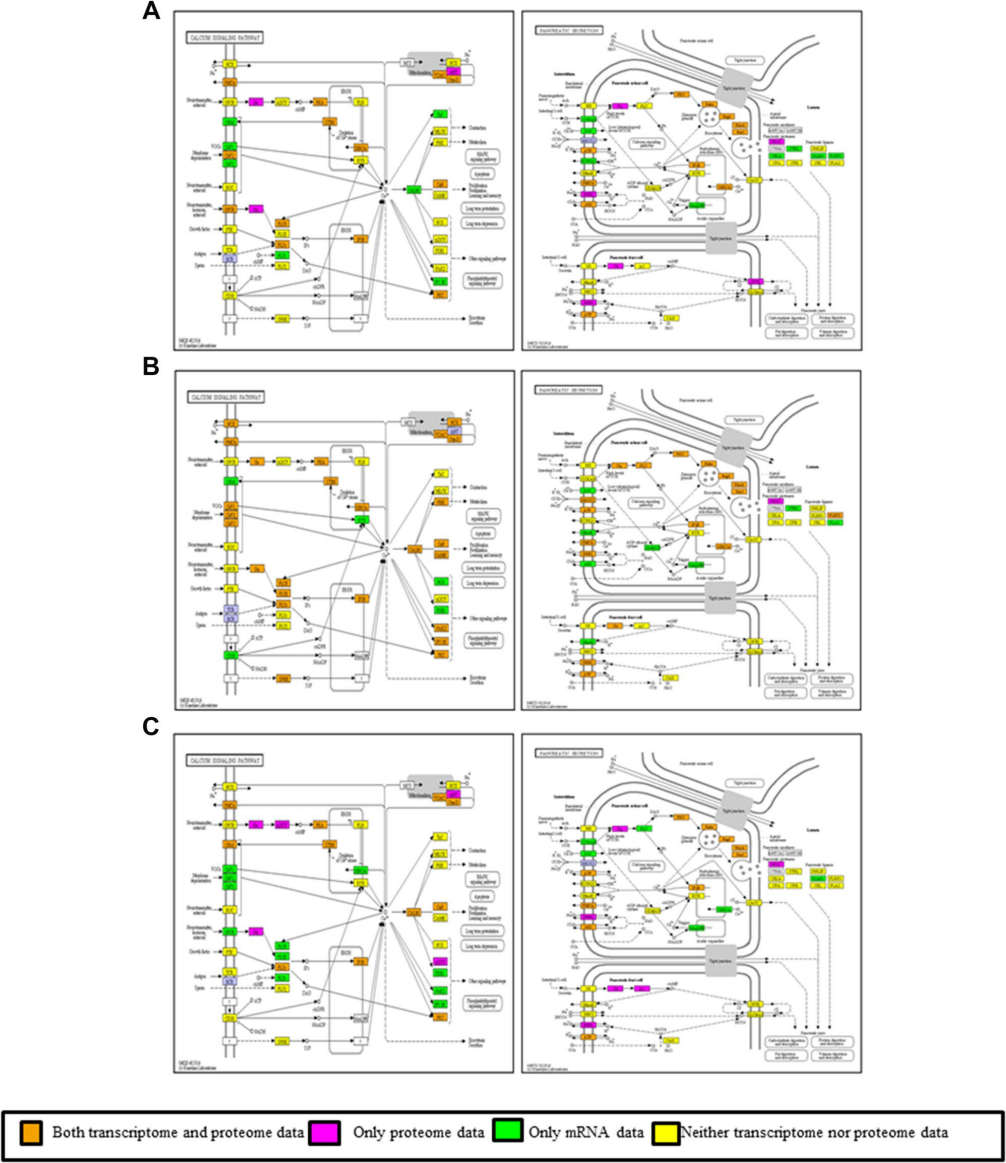
KEGG analysis of calcium signaling and pancreatic secretion pathways for (**A**) M-CHO (**B**) SP2/0 (**C**) CHO-K1 cell lines using proteomics and transcriptomics analysis. Orange stands for transcriptome and proteome data, magenta stands for only proteome data, green stands for only mRNA data and yellow stands for neither transcriptome nor proteome data (https://www.kegg.jp/kegg/kegg1.html).

A heat map for a group of proteins found to be more depleted in CHO cells compared to SP2/0 cells was generated in Fig. 3B. For example, glycosphingolipid biosynthesis, ABC transporters, PPAR signaling, calcium signaling, cell adhesion molecules, mucin-type O-glycan biosynthesis, and secretion associated pathways, were found to be under-represented in both CHO-K1 and M-CHO cell lines compared to the SP2/0 cells, with calcium signaling and pancreatic secretion selected for further analysis. Since the pancreas has the highest protein synthesis rate in mammalian organs^23^, we were especially interested in looking for the differences between SP2/0 cells, coming from mouse spleen, and CHO cells. The KEGG pathway analysis of calcium signaling and pancreatic secretion in Fig. 4 helped to further elucidate potential functions under-represented in CHO cells. SPHK2, CD38, Slc8a and many other genes involved in calcium signaling were not detected in either deep sequencing transcriptomic or proteomic analysis for both M-CHO and CHO-K1, while these genes were present in proteomics and/or transcriptomics data sets of SP2/0. Calcium signaling is a versatile signaling network affecting a wide range of cellular functions, including gene transcription, cell proliferation, secretion and exocytosis^24^, and the importance of calcium signaling, both in endocrine and exocrine secretory cells has been previously demonstrated^25^. Pla2, a calcium-dependent lipase associated with phospholipid remodeling of bio-membranes in many cell types, and MaxiK (large conductance, voltage and calcium sensitive potassium channel), which plays a key role in regulating calcium-sensitive potassium channels for membrane potential and is important to exocytosis, mapped to the SP2/0 but were depleted in M-CHO and CHO-K1. This result is not unexpected since calcium signaling is important to the development and function of B cells^26^.

### Functional analysis of CHO and SP2/0 cell lines

**Figure 5:**
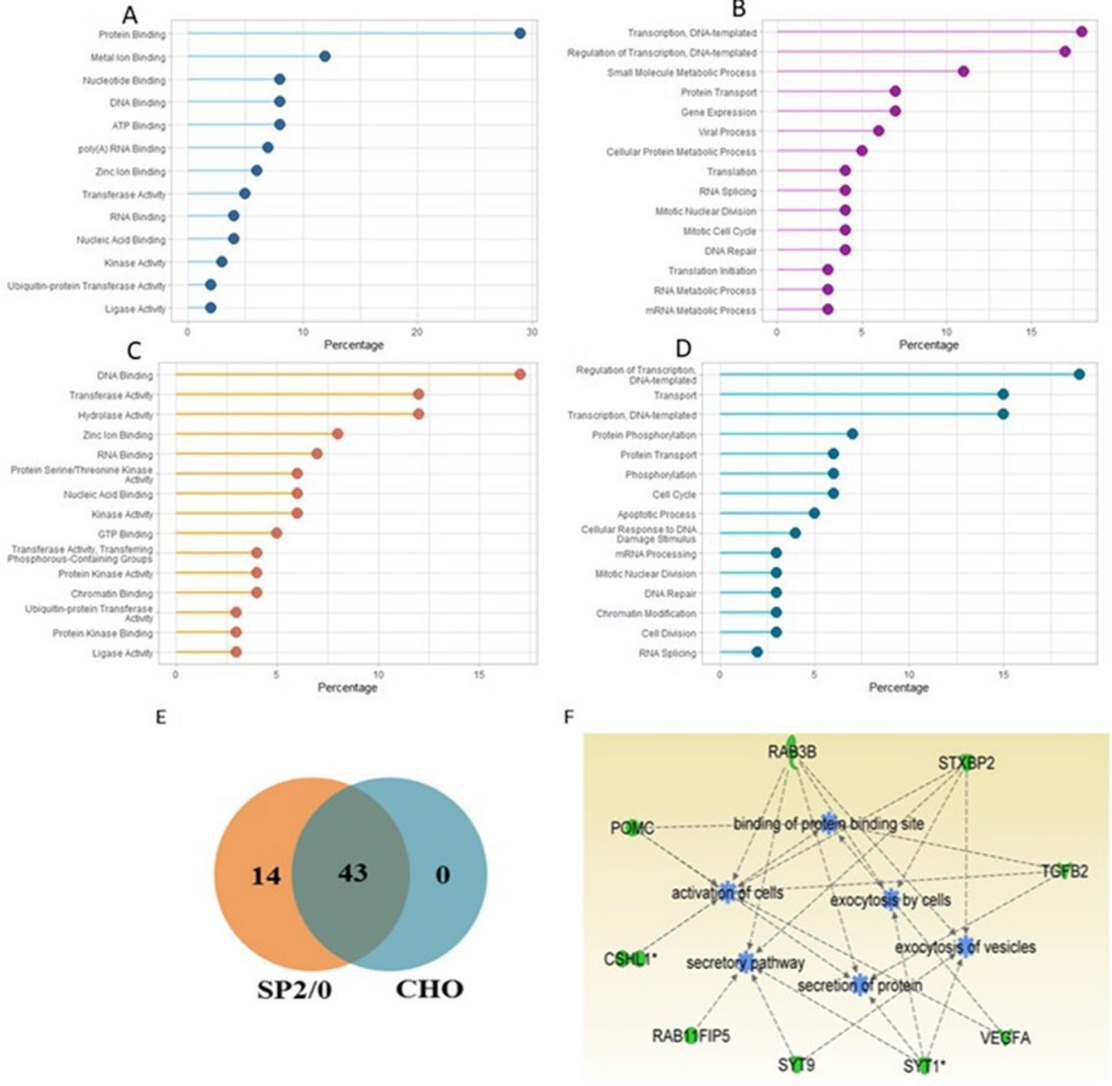
Enrichment results of gene ontology analysis of (**A**) M-CHO molecular function (**B**) M-CHO biological process (**C**) SP2/0 molecular function (**D**) SP2/0 biological process (**E**) Secretory granule genes present in SP2/0 and CHO cells (**F**) Ingenuity Pathway Analysis of secretory granule genes present in SP2/0 cells.

In order to gain a better understanding of the biological process (BP), molecular function (MF) and cellular component (CC) of the transcriptomic and proteomic profiles of M-CHO and SP2/0 cells, gene ontology (GO) analysis was implemented to identify over-represented (enriched) and under-represented (depleted) categories. The GO-CHO database was used, and enrichment and depletion *p*-values for MF, BP and CC were found and are listed in Supplementary Tables 9 to 11^27,28^. The enrichment results of the M-CHO and SP2/0 cell lines are summarized in Fig. 5. DNA and RNA binding, ubiquitin transferase activity and ligase activity were observed among the top 15 enriched molecular functions in both M-CHO and SP2/0 cells. Alternatively, biological processes, such as transport, phosphorylation, and apoptosis were more enriched in SP2/0 cell lines. In terms of depleted biological process, signal transducer activity and G-protein-coupled receptor activity were among the top 15 depleted pathways of both M-CHO and SP2/0 cells, while ion and potassium channel activities and cell to cell signaling were more depleted in M-CHO.

Interestingly, the integral components of membrane, cell surface and plasma membrane terms, and secretory granules were found to be under-represented for the CC analysis in M-CHO cells and enriched in SP2/0 cells. Individual genes representing the secretory granule category were compared between the SP2/0 and CHO cells with the resulting overlap shown in Fig. 5E. The eighteen genes, found only in the SP2/0 cell data, were then subjected to Ingenuity Pathway Analysis (IPA). Interestingly, proteins such as RAB3B, SYT1, SYT9 and RAB11FIP5 involved in secretion of proteins and vesicle exocytosis were found in SP2/0 data but were missing from the M-CHO data, as shown in Fig. 5F and Table S12.

### CHO membranome exposure

**Figure 6:**
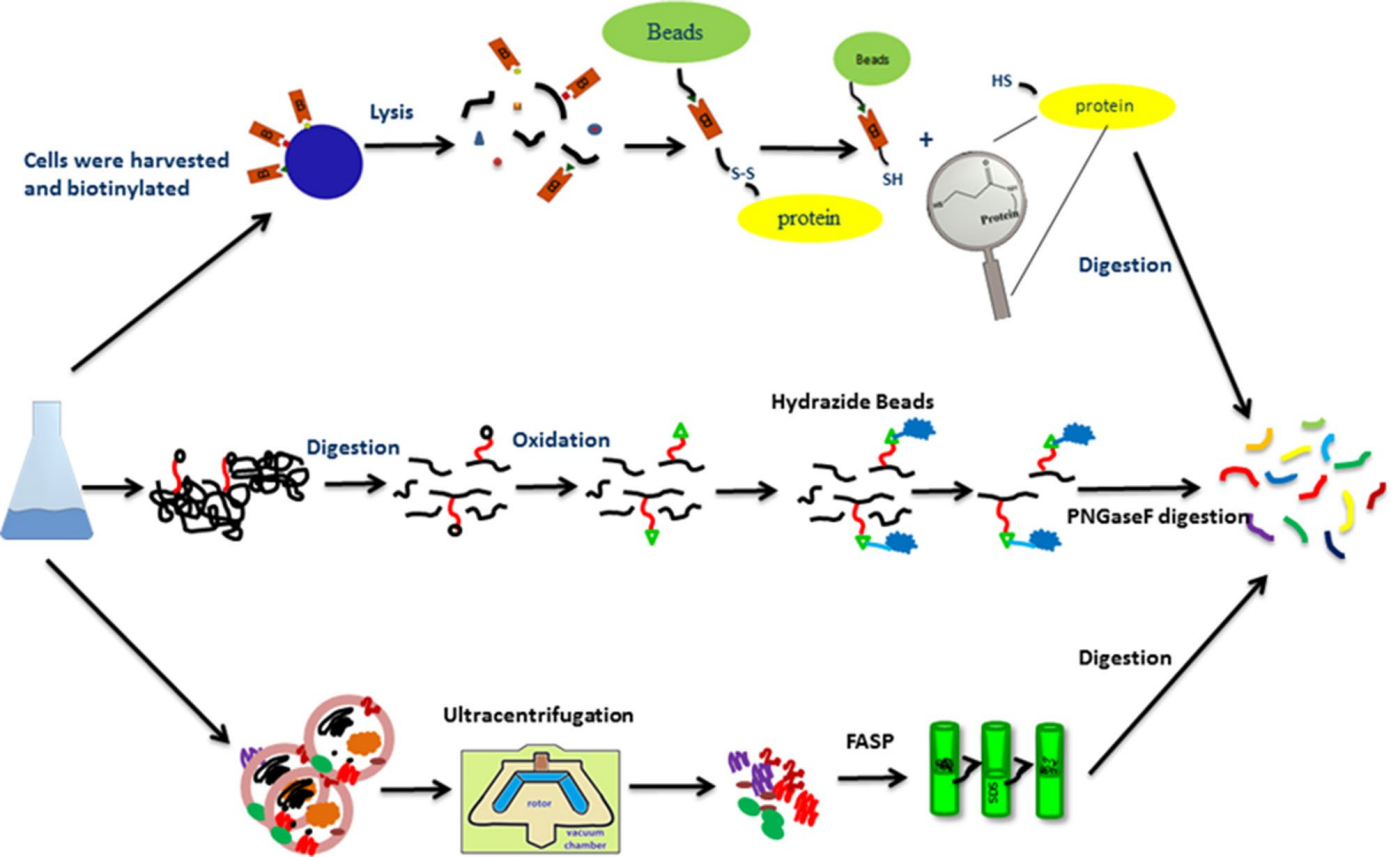
CHO membranome analysis with cell surface biotinylation, glycoproteome and two step ultracentrifugation based enrichment methods.

Both transcriptomics and proteomics data, gene ontology and KEGG pathway analysis revealed that membrane or secretion associated pathways were often depleted in M-CHO or CHO-K1 cells, whereas these pathways were enriched in SP2/0 cells. Membrane biogenesis is known to be enriched in murine cells, but these findings also suggest that this category of proteins may also be low in M-CHO cells^29,30^. Although M-CHO cells are widely used both for secreted and membrane protein expression, poor expression of membrane proteins has been previously reported^31^. In order to further examine the presence of key membrane and vesicle proteins in M-CHO cells, we applied three different enrichment methods to explore the M-CHO membranome. Two step ultracentrifugation, cell surface biotinylation and hydrazide chemistry-based glycoproteome enrichment methods were coupled with LC/MS/MS as shown in Fig. 6 to evaluate both membrane and secretory vesicle proteins. While cell surface biotinylation identifies plasma membrane proteins, glycoproteome enrichment identifies proteins traveling through the ER and Golgi apparatus along the secretory pathway. The two step ultracentrifugation technique based on sucrose and NaCO_3_ treatments allowed for the isolation of the vesicular proteome, exosome and plasma membranome.

The unique peptide numbers and protein groups for each analysis are summarized in Table 2, and the data for glycoproteome, ultracentrifugation and biotinylation can be found in Supplementary Tables S13, S14 and S15, respectively. The proteins from each isolation were subjected to a variety of bioinformatics tools, including TMHMM^32^, SignalP^28^, TargetP^33^, Phobius^34^, and WolfPSort^35^ in order to identify those containing transmembrane domains and/or signal peptides^27^. Although glycoproteome enrichment provided the highest percentage of either membrane or secreted proteins, the ultracentrifugation-based membrane proteomics technique revealed the highest number (1483) of membrane and/or secreted proteins. For this reason, peptides from the ultracentrifugation enrichment were separated into 48 fractions using bRPLC followed by tandem mass spectrometry analysis. Coupling enrichment technology with the two-dimensional fractionation technique identified 86,646 peptides belonging to 8736 proteins, with an average of 10 peptides per protein. Of these proteins, 2478 were predicted to be on the membrane, based on WolfPsort, TMHMM and Phobius-TM, whereas 2804 were predicted to be secreted, based on WolfPsort, SignalP, TargetP and Phobius-SP (Supplementary Table 16.), while some were predicted to be both membrane and secreted. As a result, approximately 47% (or 4160) of the total proteins identified were predicted to be either membrane and/or secreted. When we combined all the proteins from the cell proteome, glycoproteome, cell surface biotinylation and ultracentrifugation experiments, the number of total proteins increased to 9941, with membrane enrichment work described above identifying an additional 1889 proteins. Furthermore, of these 1889 proteins, 529 were not found in RNAseq data. GO cellular component analysis of these newly elucidated membrane-associated proteins found that 68% of the proteins identified in M-CHO were found to be localized either on the membrane or extracellular space, including important vesicular transport genes such as Ap3b2, A2m, and Srebf2, along with Rab proteins such as Rab33a, Rab40b, Rab19, Rab11fip2. However, when we mapped the newly identified proteins from the membranome to the secretory granule pathway, we were only able to identify BRCA2 out of the 16 secretory granule proteins listed in Supplementary Table 12.

**Table 2:** Summary of ultracentrifugation, cell surface biotinylation and glycoproteome enrichment coupled mass spectrometry results and membranome analysis.

Method	Unique peptides	Protein groups	Transmemb. domain and membrane	Signal peptide and extracellular	Membrane & extracellular
Ultracentrifugation based enrichment	11,099	2525	1076	905	1483
Glycoproteome enrichment	961	466	347	310	449
Biotinylation enrichment	11,751	2306	624	736	1060

Even after the secondary membrane proteomics experiments, many of proteins and pathways associated with secretory and membrane pathways were still depleted in CHO cells compared to SP2/0 cells, with depletion values listed in Supplementary Table 17. Thus, most of the membrane and vesicle proteins appear to remain in low abundance in CHO cells even after these secondary isolation approaches.

## Discussion

A comprehensive systeomics analysis was undertaken to elucidate and compare the physiology of CHO and SP2/0 cell lines. This approach combined both transcriptomics and proteomics profiling together with statistical and bioinformatic analysis methods to explore the under-represented and over-represented pathways of two of the most important biopharmaceutical production cell lines. Although the mouse genome has been widely studied in literature, few reports have characterized the SP2/0 cell line using omics profiling. Coupling fractionation and enrichment technologies together with LC/MS/MS allowed us to catalog low abundant proteins in CHO cells. In concert, KEGG and GO bioinformatic tools helped categorize CHO and SP2/0 proteins based on their functions and pathways. When the hypergeometric distribution test was applied to compare exponential and stationary phases, multiple pathways, including those for apoptosis, RNA degradation, and the proteasome, displayed a higher representation in the stationary phase of the CHO cells. Increases in such activities are often associated with a transition from growth to death phases. Alternatively, when comparing CHO to SP2/0 in both phases using the transcriptomics and proteomics data sets, calcium signaling, membrane associated terms, secretory granules, and secretion associated pathways were found to be depleted in CHO cells during both phases. Indeed, these pathways are known to be highly active in B cells^26,36,37^, and SP2/0 cells were created as a fusion of spleen and myelomas while CHO cells were derived from the regions around the hamster ovary. Calcium signaling, membrane biogenesis, phospholipid synthesis, and secretory activities are critical components of the spleen activity. Similarly, retinoic acid synthesis is critical to ovary function, so an amplification of this pathway would be expected in CHO^38^.

Since proteins residing on the membrane or functioning in the secretory pathway are often in low abundance when using conventional cell isolation methods, glycoproteomics, cell surface biotinylation and ultracentrifugation-coupled membrane enrichment methods were followed by LC/MS/MS analysis to isolate and identify them in CHO. Bioinformatics tools such as TMHMM, SignalP, TargetP, and Phobius and GO helped to further elucidate the secretory and vesicle compartmental microenvironment of CHO cells. Even after enrichment, depletion tests verified the absence of membrane and secretory granule proteins in CHO cells. For example, only 1 out of 16 secretory granules were identified by membrane enrichment experiments. Proteins representing genes such as Stxbpt2, Syt1, Syt9 and Cma1 were still not found by secondary isolation, although they were noted in SP2/0. The importance of Stxbpt2 in intracellular membrane trafficking and functioning of SNAREs (soluble NSF attachment protein receptor) for membrane fusion and vesicular transport is known^39^ while synaptotagmins (Syt1 and Syt9) present on the synaptic vesicles serve as calcium sensors for exocytotic processes. Interestingly, in most secretory systems, exocytosis is often initiated by increases in the calcium concentration inside the cell^40^, and it is worth noting that calcium signaling mechanisms were depleted in CHO. These examples illustrate the capacity of systeomics to serve as a worthwhile starting point in order to discover and characterize genes and pathways that are deficient in particular production hosts such as CHO. We believe that systeomics serves primarily to elucidate why particular cell lines such as SP2/0 or CHO are efficient at certain tasks such as survival or secretion. Secondly, we believe efforts such as those described here can serve as a key launching point for subsequent synthetic biology and metabolic engineering interventions aimed at generating engineered cell lines with improved properties such as cell growth, protein folding, vesicular transport and secretion for biopharmaceutical applications.

## Methods

### Cell batch culture

A proprietary AstraZeneca CHO cell line was cultured in CD-CHO (Life Technologies, USA) medium and supplemented with 6 mM L-glutamine (ThermoFisher Scientific, Waltham, MA). SP2/0 cells were cultured in EX-CELL® Sp2/0 Serum-Free Medium with 8 mM L-glutamine (Millipore Sigma, Burlington, MA). CHO-K1 cells used in this study are the adherent cells grown in serum containing media^11^. Both cell lines were grown in shaking incubators at 37 °C/5% CO2/120 rpm. A Vi-cell TMXR cell Viability analyzer (Beckman Coulter, Brea, CA) was used for cell counting.

### RNAseq methods

A total of three biological replicates were undertaken for the transcriptomics studies. Qiagen RNeasy kit was used to extract the mRNA from each cell line using the manufacturer’s protocol (Qiagen, Germantown, MD). After assessing the quality and quantity of mRNA with a Nanodrop spectrophotometer (ThermoFisher, Waltham, MA) and Agilent Bioanalyzer (Santa Clara, CA), Poly A was depleted. Illumina’s Truseq RNA preparation methodology was used to prepare RNAseq libraries according to the manufacturer’s suggested protocol. Agilent DNA High Sensitivity Bioanalyzer and Library quantitation qPCR kit (Kapa Biosystems, Cape Town, South Africa) was used to assess the quality and quantity of the RNAseq libraries. Illumina HiSeq 2000 was used for library sequencing using a 100 bp paired end sequencing strategy. A TruSeq RNA Sample Preparation kit (Illumina, San Diego, CA) was used to prepare RNA libraries, and the paired-end (2 × 75 bp) reads were sequenced on a HiSeq 2000.

### CHO RNA-seq data analysis

The reference sequences for CHO cell lines were obtained from the NCBI RefSeq database, which has a total of 109,151 contigs. The N50 size of these contigs was 502 bp, and the total size was 2,399,770,464 bp. Contigs <  = 5 kb in length were discarded for more accurate analysis. The retained 9,020 (9.0%) contigs, with a total length of 2,330,772,269 bp (97.1%), were used for further analysis. The Tophat2 program was used to map the RNA-seq reads to the CHO reference sequences^41^. HTSeq (http://www-huber.embl.de/users/anders/HTSeq/) was used for counting the number of mapped reads per gene and eliminating ambiguous alignments. The count table was normalized with a DESeq package from Bioconductor.

### Proteomics

Cells were washed with cold PBS at 4 °C prior to lysing in 2% SDS supplemented with 0.1 mM PMSF and 1 mM EDTA solution at pH 7.6. After sonication, a BCA assay was done to measure the protein concentration. The required protein amount was reduced with 4.5 mM TCEP at 60 °C and alkylated with 8.5 mM MMTS at room temperature for 30 min. All the samples were incubated with 9 M sequanol grade urea for 1 h at room temperature. To remove the SDS, 10 kDa FASP filters were used. The samples were then digested in FASP tubes with Trypsin/LysC (Promega, Madison, WI) enzymes at 37 °C. The dried peptide samples were subjected to bRPLC (basic reversed phase liquid chromatography) fractionation.

### Membranome enrichment methods

For the biotinylation experiment, M-CHO cells were harvested, washed and biotinylated with a Pierce Cell surface biotinylation kit (ThermoFisher, Waltham, MA) according to the manufacturer’s protocol. After elution, the proteins were subjected to the FASP method and digested with Trypsin/C-Lys-C Mix (Promega, Madison, WI) shaking at 37 °C overnight. For *N*-glycopeptide capture, the samples were reduced, alkylated and digested before oxidization with 10 mM sodium periodate (Bio-Rad, Hercules, CA) at room temperature in the dark for 1 h. The peptides were mixed with AffiPrep Hz Hydrazide beads (Bio-Rad, Hercules, CA) and 100 mM aniline (Sigma-Aldrich, St. Louis, MO) and left at room temperature gently shaking overnight. After washing away the non-glycosylated peptides, the glycopeptides attached to the beads were digested in 25 mM ammonium bicarbonate with 3 µL PNGase F (New England Biolabs, Ipswich, MA) at 37 °C, as shown previously by Zhang et al.^42^. The released glycopeptides were analyzed by LC–MS/MS. M-CHO cells were also subjected to the ultracentrifugation technique for membrane proteome enrichment. The cells were lysed in a sucrose buffer (0.24 M Sucrose, 25 mM NaCl, 50 mM HEPES, 1 mM EDTA; pH7) and centrifuged at 50,000 rpm for 96 min. Initial ultracentrifugation pellets were brought up in a resuspension buffer (0.1 M Na_2_CO_3_ and protease inhibitor; pH 11) before a second ultracentrifugation spin. Final membrane-enriched pellets were resuspended in sonication buffer in 2% SDS and subjected to the FASP method before LC/MS/MS analysis.

### bRPLC and LC/MS/MS

Digested peptides were fractionated on a basic reversed phase column (XBridge C18 Guard Column, 5 µm, 2.1 × 10 mm XBridge C18 Column, 5 µm, 2.1 × 100 mm). Fractions were merged into final 24 or 48 sample groups prior to LC/MS/MS analysis. Tandem mass spectrometry analysis of the peptides were carried out on the LTQ-Orbitrap Velos (ThermoFisher, Waltham, MA) attached to an Eksigent nanoflow 2D liquid chromatography system with an auto sampler (Eksigent, Dublin, CA). Peptides were enriched on a 2 cm trap column (YMC gel ODS-A S-10 µm), fractionated on a Magic C18 AQ, 5 µm, 100 Å (Michrom Bioresources, Auburn, CA), 75 µm × 15 cm column and electrosprayed through a 15 µm emitter (New Objective, Woburn, MA). The reversed-phase solvent gradient consisted of solvent A (0.1% formic acid) with increasing levels of solvent B (0.1% formic acid, 90% acetonitrile) over a period of 90 min. The LTQ Orbitrap Velos was set at 2.0 kV spray voltage, full MS survey scan range of 350–1800 m/z, data dependent HCD (higher energy collision dissociation) MS/MS analysis of top 10 precursors with minimum signal of 2,000, isolation width of 1.9, 30 s dynamic exclusion limit and normalized collision energy of 35. Precursor and the fragment ions were analyzed at 60,000 and 7500 resolutions, respectively. All the raw data has been deposited to NIST.

### Proteomic data analysis

Peptide sequences were identified from isotopically-resolved masses in MS and MS/MS spectra extracted with and without deconvolution using a Thermo Scientific MS2 processor and Xtract software. Data was searched against all entries in the *Cricetulus griseus* database for CHO cell lines and *M. musculus* database for SP2/0 cell lines (These databases were downloaded on the 13 Aug 2014. The total entries for Cricetulus griseus proteome used was 21,610 and the total entries for *M. musculus* used was 34,084), with oxidation on methionine (variable), deamidation NQ (variable), phosphoSTY (variable), and methylthiomethane on cysteine (fixed) as modifications, using Mascot software interfaced in the Proteome Discoverer (http://portal.thermo-brims.com) workflow. Mass tolerances on precursor and fragment masses were 15 ppm and 0.03 Da, respectively. Data was analyzed using Proteome Discoverer 1.4 software. In addition to this, CHO-K1 mass spectrometry raw data was compiled from the study used in Baycin et al. 2012. All the MS raw data was reannotated with the same strategy as M-CHO cells. 1% FDR (false discovery rate) was used for both peptides and proteins identification.

### Statistical and pathway analysis

The NSAF and FPKM values were calculated for protein and mRNA values and were compared and plotted using TIBCO Spotfire 3.1. In this current study, NSAF method was applied due to its capability of providing high reproducible data on the quantification of proteins^43,44^ compared to distributed normalized spectral abundance^45^, normalized spectral index^46^, and exponentially modified protein abundance index^47^.

Fold changes (FC) were used as selection criteria to identify candidate individual proteins of interest and to explore enriched canonical pathways along with protein/gene networks in the Ingenuity Pathway Analysis Software (http://www.ingenuity.com/). The data from all the cell lines were annotated with the Gene Ontology (GO) molecular function, biological process and cellular component categories. For GO annotation of the CHO genes, GO Cross Homology was obtained using GOCHO platform version ’14-04,’ which is publicly available at http://ebdrup.biosustain.dtu.dk/gocho. The Mouse Genome Informatics database was accessed on 11 June 2014 to download corresponding GO terms of mouse genes for SP2/0 cell line (ftp://ftp.informatics.jax.org/pub/reports/index.html#go)^48^. The Kyoto Encyclopedia of Genes and Genomes (KEGG) database pathways were downloaded from the KEGG website (http://www.genome.jp/kegg/) on 11 June 2014 for mouse and Chinese hamster species^49,50^ All calculations and programming tasks were performed using MATLAB version 2010a and R software^51^. Enrichment and depletion p-values are the outcome of a hypergeometric distribution calculated using MATLAB’s hygecdf and hygepdf functions. Adjusted p-values, Bonferroni correction was used in this study. Genesis software (release 1.7.6) was used for making heatmaps^51^. KEGG pathway mapper was used for calcium signaling and pancreas secretion pathways coloring^52^.

## Supplementary Information

Supplementary Information 1.

Supplementary Information 2.
