# Supplementary material for: Comparative systeomics to elucidate physiological differences between CHO and SP2/0 cell lines
Source: Sci Rep. 2022 Feb 28;12:3280. doi: 10.1038/s41598-022-06886-1 (PMC8885639; doi:10.1038/s41598-022-06886-1)
Supplement: Supplementary file 1 — Supplementary Information 1. [file 41598_2022_6886_MOESM1_ESM.pptx]

## Slide 1
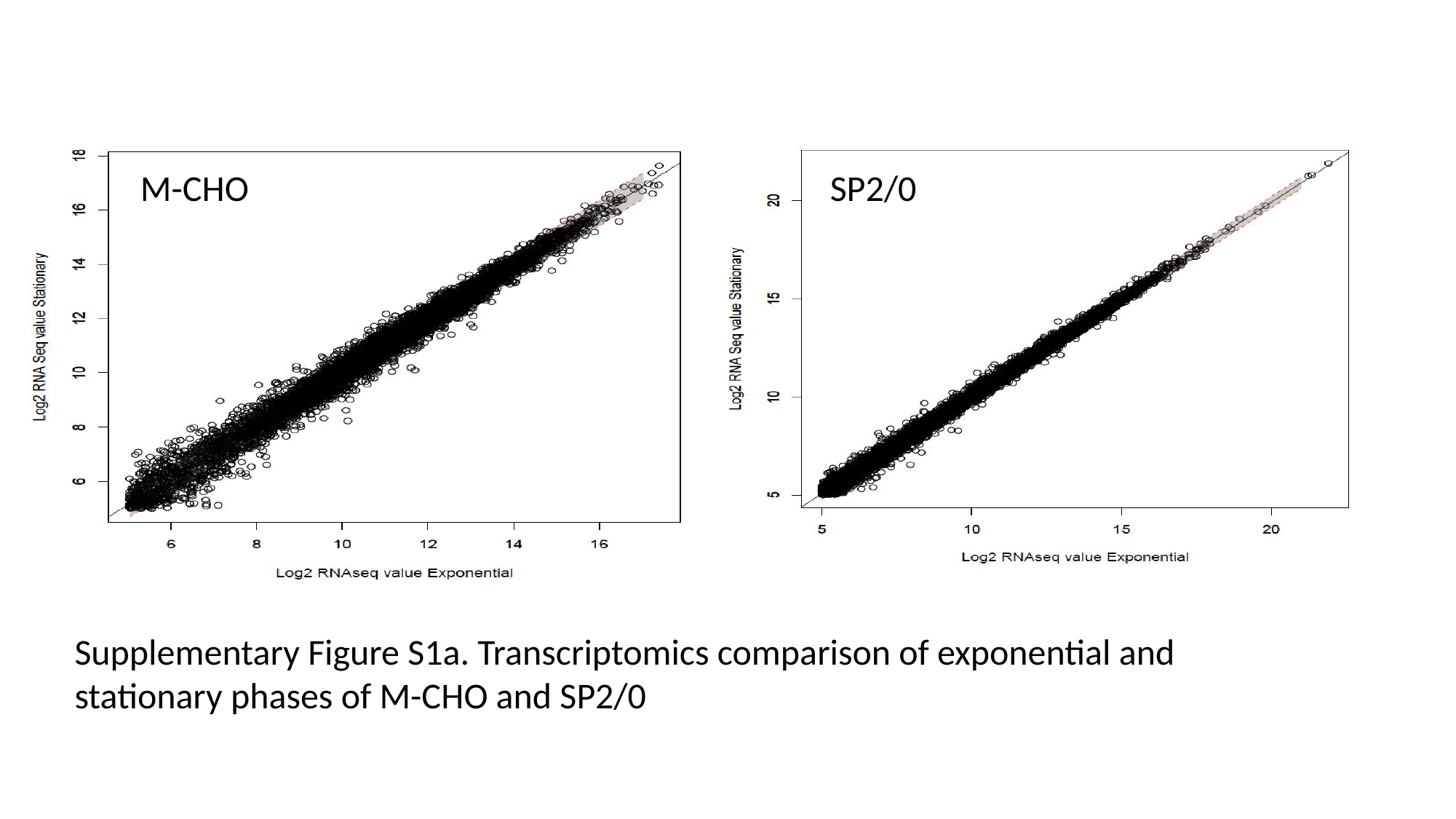

SP2/0
M-CHO
Supplementary Figure S1a. Transcriptomics comparison of exponential and stationary phases of M-CHO and SP2/0

## Slide 2
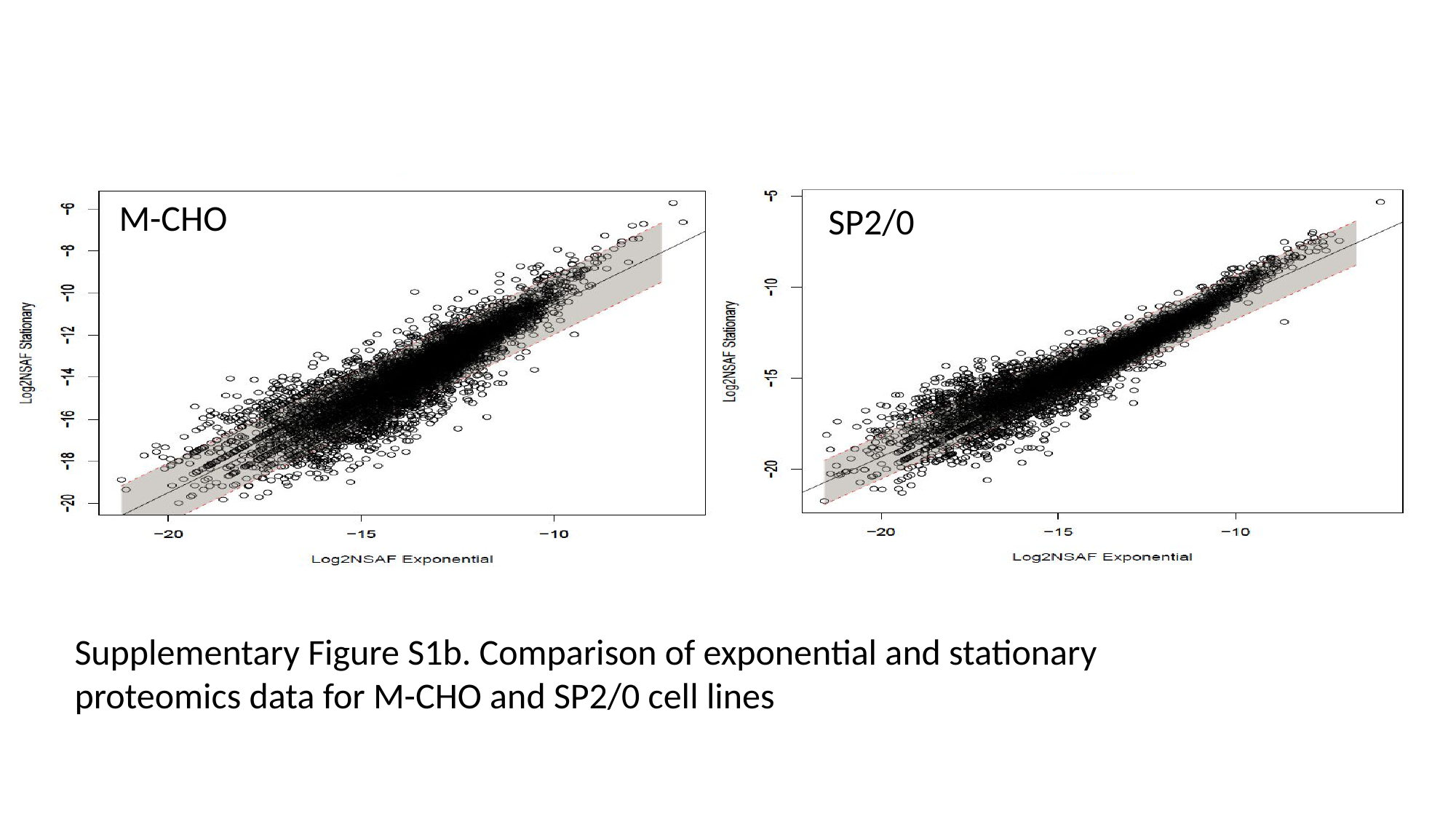

M-CHO
SP2/0
Supplementary Figure S1b. Comparison of exponential and stationary proteomics data for M-CHO and SP2/0 cell lines

## Slide 3
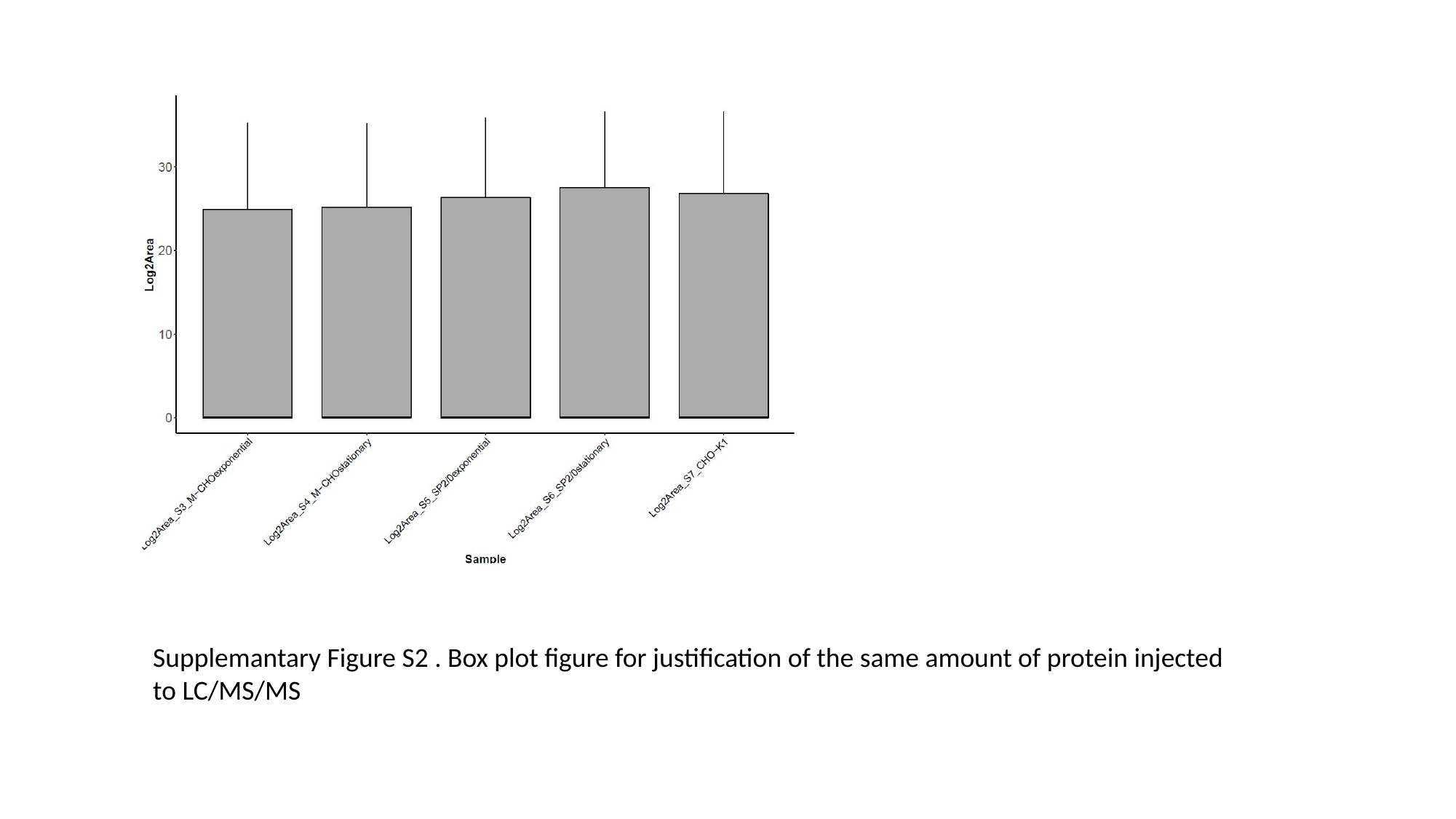

Supplemantary Figure S2 . Box plot figure for justification of the same amount of protein injected to LC/MS/MS

## Slide 4
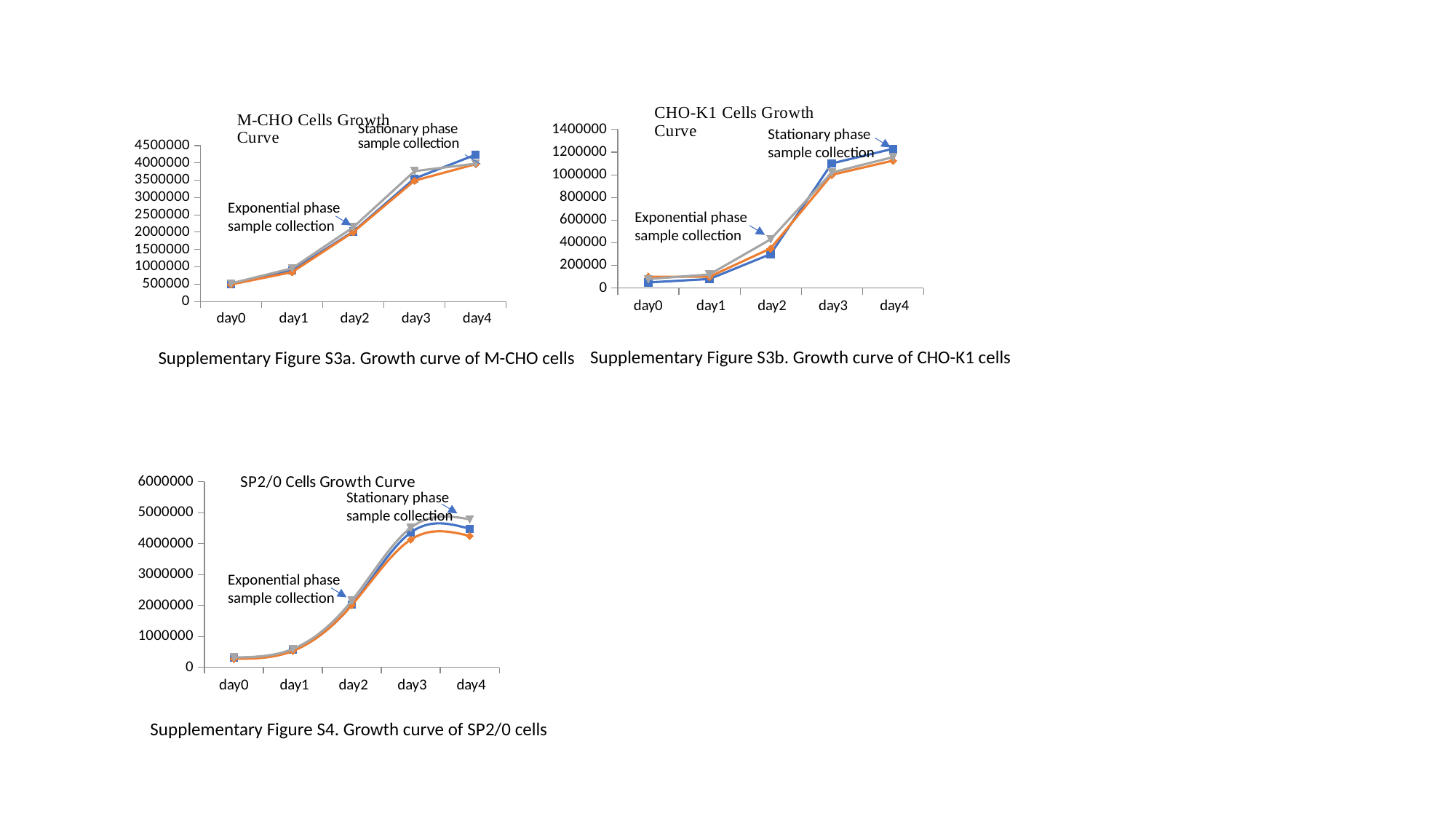

### Chart
| Category | day0 | day0 | day0 |
|---|---|---|---|
| day0 | 50000.0 | 100000.0 | 80000.0 |
| day1 | 80000.0 | 100000.0 | 120000.0 |
| day2 | 300000.0 | 350000.0 | 430000.0 |
| day3 | 1100000.0 | 1000000.0 | 1020000.0 |
| day4 | 1230000.0 | 1125000.0 | 1156000.0 |Stationary phase sample collection
Exponential phase sample collection
### Chart
| Category | CAT-S Cell Growth Curve | | |
|---|---|---|---|
| day0 | 500000.0 | 490000.0 | 520000.0 |
| day1 | 896999.9981880188 | 846789.0 | 958300.0 |
| day2 | 2021400.0774383547 | 2006348.0 | 2145754.0 |
| day3 | 3549500.0911712646 | 3487634.0 | 3763754.0 |
| day4 | 4237568.82681274 | 3965787.0 | 3978467.0 |Exponential phase sample collection
Supplementary Figure S3b. Growth curve of CHO-K1 cells
Supplementary Figure S3a. Growth curve of M-CHO cells
### Chart
| Category | SP2/0 Cell Growth Curve | | |
|---|---|---|---|
| day0 | 300000.0 | 275000.0 | 317500.0 |
| day1 | 567599.9952316284 | 527845.0 | 592350.0 |
| day2 | 2021400.0774383547 | 2008250.0 | 2153468.0 |
| day3 | 4352899.892425537 | 4123800.0 | 4506789.0 |
| day4 | 4474099.977111816 | 4245302.0 | 4783678.0 |Stationary phase sample collection
Exponential phase sample collection
Supplementary Figure S4. Growth curve of SP2/0 cells

## Slide 5
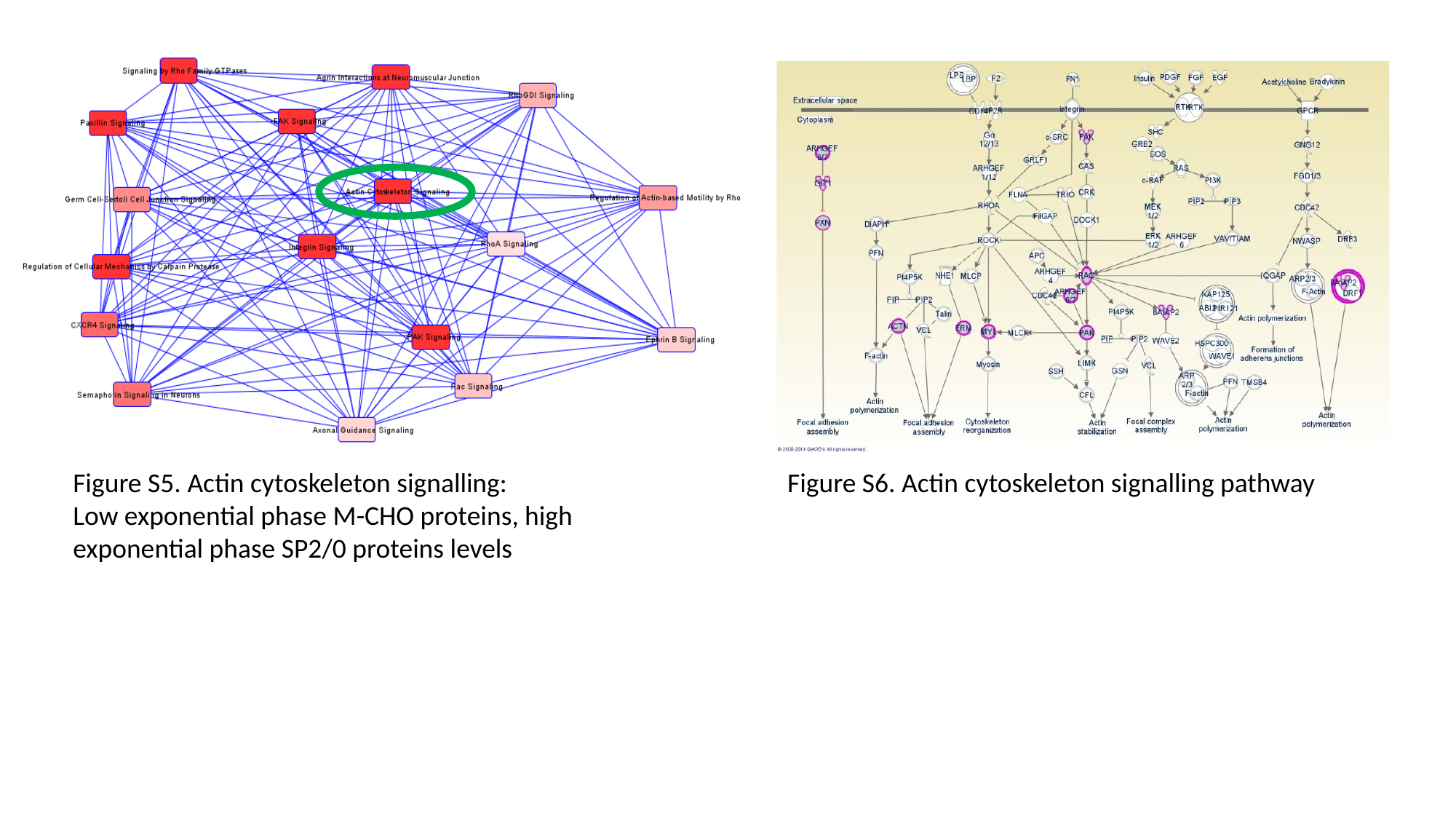

Figure S5. Actin cytoskeleton signalling:
Low exponential phase M-CHO proteins, high exponential phase SP2/0 proteins levels
Figure S6. Actin cytoskeleton signalling pathway
